# Molecular Modeling of the Pathogenetic Mechanisms of Neuropsychiatric Disorders

**DOI:** 10.3390/ijms27083563

**Published:** 2026-04-16

**Authors:** Amal Abdurazakov, Dmitrii A. Abashkin, Ekaterina V. Semina, Yulia A. Chaika, Vera E. Golimbet

**Affiliations:** Mental Health Research Center, Kashirskoe Sh. 34, Moscow 115522, Russia; aabdurazakov@edu.hse.ru (A.A.); e-semina@yandex.ru (E.V.S.); golimbet@mail.ru (V.E.G.)

**Keywords:** molecular dynamics simulation, in silico mutagenesis, neuropsychiatric disorders, molecular psychiatry, genetic variants, post-translational modifications, psychopharmacology, drug design, protein dynamics, computational biology

## Abstract

Neuropsychiatric diseases are characterized by complex molecular underpinnings that remain challenging to fully elucidate. Molecular dynamics (MD) simulations have emerged as a powerful computational tool, providing a crucial bridge between static genetic data and the dynamic functional consequences of molecular alterations. This review offers a comprehensive overview of the application of MD simulations in studying the molecular basis of neuropsychiatric disorders. We highlight key applications, including the assessment of mutation pathogenicity in disease-associated proteins, the influence of post-translational modifications on protein function, folding, misfolding, and aggregation, and the characterization of psychopharmacological drug–target interactions at atomic resolution. Through relevant examples from research on psychiatric and neurodegenerative diseases, we illustrate how these computational methods are implemented to gain mechanistic insights. Importantly, this review traces the historical development of MD simulations in biological applications, critically examines the method’s limitations, and outlines future perspectives for simulating long-timescale physiological processes, large molecular ensembles, and even whole-cell environments. Ultimately, this work highlights MD simulations as a useful and complementary tool for modern neuropsychiatry research, capable of revealing disease mechanisms and guiding the development of novel therapeutic strategies.

## 1. Introduction

Signal transmission in the nervous system exhibits multiple levels of complexity, starting from the organization of specialized cell types and cellular states into a dynamic network, and ending with highly specific protein–protein and protein–ligand interactions at the atomic resolution level. The latter interactions exhibit remarkable specificity, enabling psychoactive compounds to act at the nanomolar concentration.

Due to this high specificity, research in molecular psychiatry can benefit from bioinformatic tools capable of capturing structural states of neural proteins, as well as alterations in their structure and interactions over time at atomic resolution. Traditional techniques like cryogenic electron microscopy (cryo-EM), X-ray crystallography and nuclear magnetic resonance (NMR) provide valuable structural information, yet they are limited in their ability to comprehensively resolve the dynamic processes essential for understanding protein function in the nervous system. Cryo-EM can visualize highly ordered regions at near-atomic resolution (2–3 Å), but it offers only static or ensemble-averaged representations and cannot capture rapid conformational transitions in real time [1]. NMR spectroscopy, while powerful for probing local atomic environments and dynamic fluctuations in small to mid-sized proteins, becomes less effective for larger complexes due to spectral overlap and reduced structural resolution [2]. As neither technique alone can provide continuous, high-resolution trajectories of molecular motion, MD simulations have achieved growing recognition. They enable direct, atomistic observation of protein motions over time, allowing researchers to track conformational changes, domain rearrangements, and molecular interactions in a dynamic framework that complements and extends the static views offered by experimental methods.

The first full-scale MD simulation of a protein molecule was conducted in the late 1970s [3]. Such simulations typically begin with high-resolution structural data of the target protein or complex, most often obtained through experimental methods like X-ray crystallography, cryo-EM, or NMR spectroscopy. The effective application of MD in molecular psychiatry depends on the quality of high-resolution protein structural and mutational data. In recent years, the accuracy and coverage of protein crystal structures have significantly improved. Advanced crystallization strategies, such as microseed matrix screening [4] and high-throughput techniques [5], have facilitated the structural characterization of previously intractable targets, including membrane proteins, viral assemblies, and protein–ligand complexes. In parallel, the incorporation of machine learning and artificial intelligence has refined key stages of structural biology such as cryo-EM map interpretation, atomic coordinate optimization, and *de novo* protein fold prediction, thereby providing more precise and complete input models for MD simulations [6].

Large-scale genomic screenings in psychiatric cohorts have yielded vast databases of protein and DNA variants, many of which remain unclassified in terms of pathogenicity. Key initiatives such as the Schizophrenia Exome Sequencing Meta-analysis (SCHEMA) consortium [7] and the Autism Sequencing Consortium (ASC) [8] provide exome-wide data from individuals with schizophrenia (SZ) and autism, serving as a primary source of data regarding point mutations in protein-coding sequences. The development of rapid and large-scale methods for assessing mutation pathogenicity and identifying potential therapeutic molecules in the context of polygenic diseases is further stimulated by the growing demand for scalable and accurate predictive tools [9,10]. This includes the creation of large-scale molecular dynamics databases covering the entire human proteome, which enable the modeling of not only individual protein clusters, but also whole-cell dynamics and complex molecular cascades implicated in disease pathogenesis [11,12].

Molecular simulations are increasingly integrated into structural biology research, serving both as a tool for interpreting experimental data and for guiding the rational design of subsequent experiments. In this review, we explore the application of MD simulations in molecular psychiatry and neurobiology, aiming to demonstrate the relevance of this approach to molecular psychiatric research, especially given the increasing availability of genetic data. To do so, we will first outline the common metrics used to analyze MD simulations and then discuss the application of these simulation analyses in interpreting genetic findings. To the best of our knowledge, this is the first study within the framework of translational psychiatry that examines the application of MD in the context of a pathogenetic process.

To support this narrative review, we performed a targeted, non-systematic search in PubMed and Google Scholar (last search: 15 December 2025), using combinations of terms related to MD simulations, neuropsychiatric disorders, and protein variants/post-translational modifications (PTMs). We additionally screened reference lists and used citation tracking to capture further relevant studies. This strategy was designed to ensure coverage of key methodological and biological examples rather than to provide a formal systematic review.

## 2. Molecular Dynamics Background

### 2.1. Classical Molecular Dynamics

Molecular dynamics methods are based on the principles of classical mechanics and enable the simulation of atomic and molecular systems over time. Starting from the initial atomic coordinates of proteins, lipid membranes, and other biomolecules, MD simulations compute interatomic forces and predict particle trajectories, thereby generating a detailed representation of molecular behavior at the atomic level. These calculations are based on Newton’s second law, which states that the acceleration of each particle is determined by the sum of all interatomic forces acting upon it. This framework enables the representation of molecules as collections of material points, each possessing a specific mass, position in space, and velocity, thereby contributing to the system’s dynamic behavior.

The evolution of the entire system is described by the sequential integration of the equations of motion for each particle. The net force, required to compute the particle’s motion, is derived from the potential energy, which characterizes the interactions among all particles in the system. Once the force on a particle is known, its acceleration can be computed, followed by updates to its velocity and position over time. This stepwise procedure enables the reconstruction of individual particle trajectories and, consequently, the prediction of the dynamic evolution of the entire system.

The accuracy of MD simulations critically depends on the choice of a force field—a set of mathematical models and parameters that describe the potential energy of the system as a function of atomic coordinates. It determines the magnitude and direction of the forces acting on atoms at each simulation timestep, thereby shaping their trajectories over time. In classical additive force fields, atoms are treated as essentially static spheres with fixed van der Waals parameters and fixed partial charges, meaning electronic polarization is not modeled explicitly and does not adapt to the environment [13,14]. Unlike fixed-charge models, polarizable force fields explicitly account for electronic polarization by allowing molecular charge distributions to adapt to their local electrostatic environment. This dynamic response improves the accuracy of condensed-phase electrostatics and thermodynamics, including simulations in aqueous environments—the conditions most frequently encountered when testing hypotheses in neuropsychiatry [15]. In the AMOEBA (Atomic Multipole Optimized Energetics for Biomolecular Applications) force field, this is achieved using permanent atomic multipoles combined with self-consistent induced dipoles. However, this approach comes with a substantially higher computational cost, as the induced dipoles must be calculated iteratively at each simulation timestep [15,16].

Explicit polarizability is particularly crucial in environments with strong or highly heterogeneous electric fields. Key examples include densely charged metal-binding pockets in proteins, where many-body polarization dictates Ca^2+^/Mg^2+^ selectivity, and membrane systems, where dielectric contrast and interfacial electrostatics govern ion interactions [17,18]. Furthermore, polarizability can significantly influence binding dynamics and free-energy landscapes within confined charged sites, such as anion-binding pockets in G-protein-coupled receptors (GPCR). Conversely, traditional additive force fields often remain an efficient and adequate baseline for standard conformational sampling in bulk aqueous solutions [15,17,19].

In practice, a typical MD simulation begins with the construction of a molecular system model that includes the biomolecular target (e.g., a protein), a solvent, and the ions required to neutralize the system’s net charge and establish basic ionic strength. Although protein crystallization methods have advanced, many conformations remain resistant to crystallization. Consequently, AlphaFold 2, OpenFold and RoseTTAFold enabled the *de novo* modeling of three-dimensional protein conformations directly from amino acid sequences, achieving accuracy comparable to experimental techniques [20,21,22,23,24]. The subsequent release of AlphaFold 3 and Boltz-1 scaled this technology, facilitating the prediction of joint spatial configurations of proteins, nucleic acids, ligands, and their covalent modifications within a unified computational framework [25,26].

The effective integration of these models into MD workflows is contingent upon per-residue confidence metrics, where pLDDT serves as a score reflecting how much the model trusts the local 3D structure at each position in the protein [27]. High predicted local distance difference test (pLDDT) regions serve as stable structural cores, while low-pLDDT segments typically represent intrinsically disordered regions (IDRs) or highly flexible loops common in neural signaling proteins [28,29]. Since pLDDT monitors local accuracy rather than global architecture, multi-domain arrangements must be cross-validated via Predicted Aligned Error (PAE) and PTM scores to ensure the starting topology is biophysically plausible [30,31]. This framework supports a more disciplined reading of MD trajectories, since sustained root mean square deviation (RMSD) in high-pLDDT regions are more likely to raise concerns about force-field or setup issues, whereas elevated root mean square fluctuation (RMSF) in low-confidence segments is often consistent with genuine exploration of flexible conformations within the protein’s ensemble [32].

After defining the system and protein topology, energy minimization is performed to bring the system into a stable state and eliminate any unnatural states that may have arisen during system preparation for the simulation. To approximate the physiological conditions critical for neural receptor and transporter dynamics, the molecular assembly is typically solvated in explicit water, which serves as the dominant solvent. Molecular dynamics therefore offers several refined rigid water models (TIP3P, SPC/E, and the TIP4P family), and each model trades computational efficiency against how well it reproduces core condensed-phase properties such as density, dielectric response, diffusion/viscosity, and hydrogen-bond structure [33,34,35]. Because these differences propagate to biomolecular observables, researchers usually choose the water model to match the protein force field and ion parameters, since a mismatched solvent/ion setup can bias hydration and electrostatic screening, alter salt-bridge stability and protein compactness, and shift hydration-controlled gating, as well as the conformational dynamics of transport channels, internal cavities in enzymes, and association thermodynamics, even under identical Particle Mesh Ewald (PME) settings [36,37,38,39].

In psychopharmacology research, mechanisms of passive drug permeation, lipid nanodomain formation, lipid–protein interactions, membrane curvature, and synaptic vesicle fusion are central to computational modeling [40]. Poorly constructed membrane systems, however, generate severe artifacts that skew MD results. An inadequate simulation box size introduces periodic boundary artifacts that constrain the conformational dynamics of extra- and intracellular protein domains [41]. Inaccurate lipid ratios or mismatched leaflet surface areas in asymmetric membranes drive artificial molecular aggregation, excessive differential tension, and altered bilayer mechanics [42]. Suboptimal force fields also prevent sampling algorithms from crossing high energy barriers. This introduces hysteresis when simulating polar molecules, yielding inaccurate free energy profiles for drug binding and membrane permeability [43].

**Figure 1:**
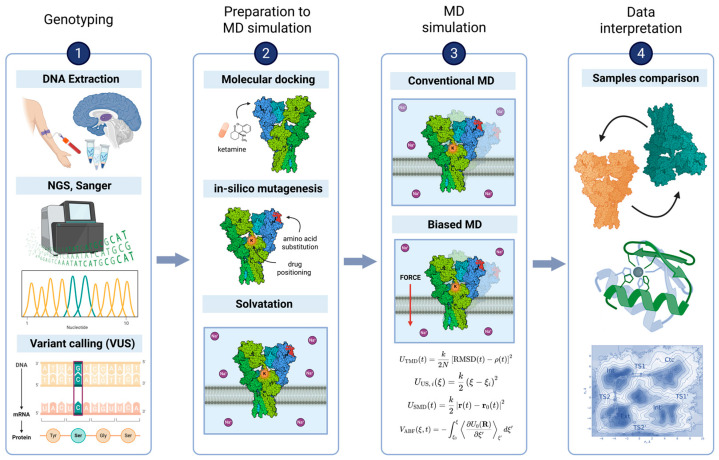
**A computational workflow for investigating the impact of a genetic or epigenetic variant.** The pipeline consists of four stages: (**1**) Genotyping to identify a variant of uncertain significance (VUS) from DNA sequencing. (**2**) System preparation for simulation, including molecular docking, in silico mutagenesis, and solvation. (**3**) Execution of conventional or biased MD simulation. (**4**) Data interpretation through analysis of structural stability (RMSD, RMSF), secondary structure, and energy landscapes. Created with BioRender, https://tinyurl.com/4whu8n79 (accessed on 12 March 2026).

**Figure 2:**
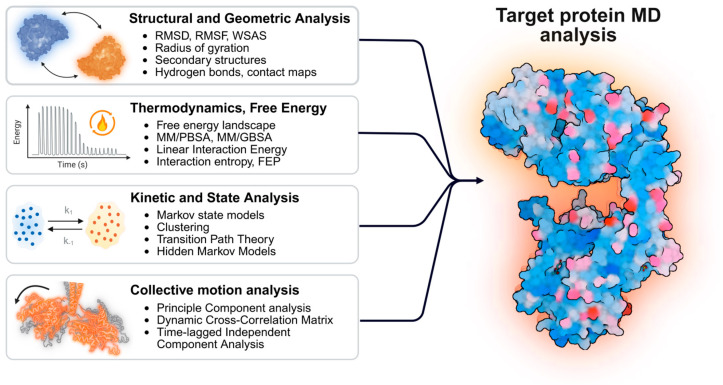
**Methods for the multi-faceted analysis of MD data**. Classical analysis is focused on four primary areas: (1) Structural and geometric analysis assesses the overall stability and local conformational changes of the protein over time. (2) Thermodynamic analysis focused on quantifying the energetics of the system, such as interaction free energies and binding affinities. (3) Kinetic and state analysis models the long-term dynamics and characterizes the transitions between different functional states. (4) Collective motion analysis aimed at identifying the large-scale, coordinated movements essential for the protein’s biological function. Created with BioRender, https://tinyurl.com/3bv6nbrd (accessed on 12 March 2026).

The simulation includes a critical phase of thermodynamic regulation involving the use of thermostats and barostats [44,45]. This stage is essential for maintaining strict control over the prescribed physical parameters of the system. Establishing such stability is a fundamental prerequisite for ensuring the reproducibility of the resulting data. This sequence of preparatory steps (Figure 1) defines the initial simulation setup by specifying the ligands interacting with the protein and selecting the starting protein conformation (e.g., open or closed). To study point mutations, variants can be introduced in silico by substituting the relevant residues and locally optimizing the surrounding geometry to obtain a physically plausible mutant structure. Once the equilibration phase concludes, the system undergoes the production simulation. The resulting output is the final MD trajectory, defined as a sequence of the coordinates of all atoms recorded at each time step, which is subsequently analyzed via established methods (Figure 2):

1. In MD simulations, a key objective is to evaluate biomolecular stability and monitor structural changes over time. This is commonly done using RMSD, which quantifies how much a structure at each time point deviates from a reference structure [46]. Increasing RMSD generally indicates larger conformational rearrangements, whereas a plateau with small fluctuations suggests equilibration. Therefore, RMSD is widely used as a primary indicator of conformational stability and simulation quality.
2. Local flexibility, in contrast, is characterized by RMSF, which complements stability metrics by quantifying the mobility of individual atoms or residues over the trajectory [47]. Regions with high RMSF typically correspond to flexible segments such as loops and termini, whereas low RMSF values indicate rigid, structurally stable parts of the protein, often including the core.

RMSD and RMSF help flag drift and flexible regions, but they are not inferential statistics and can appear stable while the underlying distribution is under-sampled because adjacent frames are correlated [48]. Interpreting these metrics credibly requires estimating effective sample size and uncertainty from decorrelated blocks and treating a plateau as only weak evidence of convergence unless it holds across independent replicas and biologically relevant timescales.

3. The radius of gyration (Rg) parameter reflects the degree of molecular compactness and allows monitoring whether the structure retains its integrity during the simulation or, conversely, undergoes expansion and unfolding. Rg and RMSF are often analyzed together to obtain a comprehensive assessment of a system’s dynamics. This approach allows for the simultaneous characterization of global disorder and changes in structural compactness via Rg, as well as the local mobility of individual atoms or amino acid residues via RMSF.
4. Principal component analysis (PCA) is used to capture the largest, functionally relevant collective motions in MD trajectories, such as open–closed transitions or domain rearrangements [49]. Typically, trajectories are first aligned to remove overall rotation/translation and a subset of atoms is selected to reduce computational cost and suppress high-frequency local noise, thereby emphasizing collective, functionally relevant motions. PCA then decomposes the covariance matrix of atomic fluctuations into eigenvectors (directions of motion) and eigenvalues (their amplitudes). The leading components (e.g., PC1/PC2) explain most of the variance and enable visualization and comparison of major conformational transitions.

PCA reduces MD trajectories to directions of maximum variance, but its output is strictly limited by the conformational states sampled during the simulation [49]. In neurobiological systems with slow dynamics, such as GPCR activation or neurotransmitter transport, short trajectories often cause PCA to misidentify random fluctuations within a single stable state as a meaningful biological mechanism [50]. Furthermore, random high-dimensional diffusion frequently produces spurious patterns that appear deceptively organized but represent nothing more than statistical noise [50,51]. Comparing mutants or ligands within a fixed PC basis is often mathematically unstable because shifts in loop flexibility or membrane composition alter the underlying coordinate system. Consequently, PCA serves primarily as an exploratory tool for hypothesis generation; it must be validated through independent replicas or replaced by state-based models, such as Markov state models (MSMs), when quantifying kinetics and state populations [50,52].

5. MSMs simplify complex molecular motion by describing it as transitions between a finite set of stable conformational states and by estimating the probabilities and rates of these transitions, for example rearrangements between active and inactive GPCR states or β-sheet growth during amyloid aggregation [53,54,55,56]. This approach is useful for rare events that are hard to observe in conventional MD because they require crossing high energy barriers and occur on long timescales, such as metastable switching or slow structural rearrangements. To construct an MSM, structures from MD are grouped into clusters using structural similarity measures such as Cα RMSD or coordinates of a reaction center, which helps identify stable conformations and quantify their contribution to overall behavior. In practice, MSM conclusions are most defensible when they remain consistent after building the model from multiple independent trajectories and after checking that the inferred kinetics do not change substantially when the lag time or the state definition is varied, since these choices can otherwise create an illusion of well-defined long-time kinetics [57].
6. The MM/PBSA and MM/GBSA methods are employed to approximate binding energetics and estimate relative changes in binding free energy and affinity in ligand–protein and protein–protein systems, including inhibitor–target interactions and mutation-related shifts in binding [58,59,60]. Complementary hydrogen-bond analyses can add mechanistic context by localizing key contacts and assessing their persistence over time. However, MM/GBSA is an end-state approximation, so its numerical values are sensitive to modeling choices such as the force field and the implicit-solvent model, and it should be interpreted primarily as a comparative tool within a consistent setup rather than a definitive measure of affinity [61].

For interpretability and reproducibility, MM/GBSA results should be reported with uncertainty estimates derived from independent replicas or statistically independent blocks, rather than from a single trajectory [61]. In routine MM-GBSA workflows, entropy is often omitted because rigorous estimation is computationally expensive, yet this can bias results and overestimate affinity when binding substantially restricts ligand or protein mobility. To reduce this interpretability gap, auxiliary entropy estimators can be added. For instance, WSAS provides a rapid geometry-based proxy by linking reduced solvent-accessible surface area to reduced conformational mobility [62], while Interaction Entropy derives an entropy proxy from MD by quantifying fluctuations in intermolecular interaction energies over time, with larger fluctuations implying greater dynamic freedom [63]. Used alongside MM-GBSA, these additions help distinguish strong interactions from strong interactions accompanied by a large entropic penalty, enabling clearer interpretation of modification-induced changes in binding.

While such end-state methods provide efficient estimates, determining precise affinity differences often requires a more rigorous thermodynamic treatment. Free energy perturbation (FEP) calculates relative or absolute binding free energies by gradually transforming one molecular state into another across a series of nonphysical intermediate states [64]. Unlike end-state approximations, FEP evaluates free energy differences along a defined thermodynamic pathway. With sufficient sampling and careful system preparation, it provides highly accurate affinity predictions for computational drug discovery [65]. In neuropsychiatric molecular modeling, FEP quantifies how point mutations, ligand modifications, or shifts in protonation and chemical substituents alter receptor or transporter binding. These thermodynamic data directly complement the structural and dynamical readouts of classical MD simulations [66].

Although rooted in classical mechanics, MD relies on inherent assumptions regarding parametrization, boundary conditions, and thermodynamic ensembles. This sensitivity is critical in neural signal transduction and protein–protein interaction. When evaluating the serotonin-gated 5-HT3 receptor, which acts as a key mediator of fast synaptic transmission, modifying water models or incorporating induced polarization fundamentally alters the hydrophobic gate’s predicted wetting and single-ion free-energy profiles. Consequently, identical structural states yield divergent conductance predictions based entirely on the underlying physical model [67]. Therefore, establishing reliable molecular etiologies for psychiatric targets dictates that computational predictions be continuously benchmarked against structural and functional experimental findings.

### 2.2. Enhanced Sampling and Biased MD Approaches

Standard MD often traps systems in local energy minima, restricting the observation of slow conformational changes such as transporter gating or receptor binding. Overcoming these barriers requires microsecond to millisecond timescales. Because these durations exceed typical simulation limits [68,69], researchers use enhanced sampling techniques to modify the potential energy surface. When the reaction coordinate is known, such as the movement of a sodium ion or GABA molecule during the alternating access cycle of GAT-1, umbrella sampling can restrain the system along that path to calculate a precise free-energy profile [70,71]. If the transition pathway is unknown, metadynamics applies a history-dependent bias to previously visited states. This forces the system to explore new conformations, mapping the broader free-energy landscape of large-scale structural shifts, such as the transition between inward- and outward-facing transporter states [72,73].

Systems with multidirectional barriers and no clear reaction coordinate require a different approach. Accelerated MD (aMD) smooths the potential energy surface by artificially reducing the depth of local minima. This allows intrinsically disordered regions, such as GPCR intracellular loops, to transition rapidly between states [68,69].

Aside from modifying the thermodynamic landscape, transitions can be driven by direct mechanical force. Targeted MD (TMD) applies time-dependent geometric restraints to pull a system toward a known target conformation. Because TMD strictly moves the biomolecule between defined initial and final states, it generates plausible transition pathways, such as C-loop closure in α7 nicotinic receptors or AMPA receptor activation, rather than extracting exact thermodynamic parameters [14,74,75].

Determining the actual physical work involved in molecular events is essential for quantifying the energetic barriers that dictate receptor affinity and transporter kinetics. To extract these thermodynamic profiles, researchers rely on steered MD (SMD). Unlike TMD, SMD applies an explicit external force to specific atoms along a pulling coordinate, typically using a virtual spring moving at a constant velocity [76]. This mechanical setup directly measures the energy required for ligand unbinding, substrate translocation, or force-induced structural rearrangements. For instance, SMD simulations have mapped the exact energetic barriers to moving dopamine and GABA through their respective transporters and have tested the conformational stability of various glutamate receptors [76,77,78].

## 3. Analysis of Significant Mutations

The position of a mutation within a single gene can critically determine the severity of the resulting phenotype, particularly in the context of neurodevelopmental disorders. Consistent with this principle, the study of pathogenic variants in ion transporters associated with epilepsy and familial hemiplegic migraine has shown that these variants are predominantly localized in residues situated near the membrane-embedded pore [79]. Mutations that affect the amino acid sequence within coding regions are particularly noteworthy, as they can impair protein function by disrupting catalytic sites in enzymes or by altering the protein’s structural integrity, dynamics, interaction profiles, or intracellular localization [80].

To move from variant localization to mechanistic interpretation, residue substitutions can be introduced in silico and examined using structure prediction and MD-based analyses. This approach was applied to model an amino acid substitution in the presynaptic protein NRXN1 using AlphaFold 2 [81]; mutations in this protein have been associated with autism spectrum disorder (ASD), Intellectual Disability (ID), and SZ [82,83,84]. Subsequently, the behavior of both the native and mutant proteins can be modeled to compare their dynamic properties, spatial architecture, and molecular interactions with ligands or other proteins. This approach enables the in silico assessment of the potential functional and structural consequences of mutations prior to conducting resource-intensive in vitro or in vivo experiments.

The originally described p.Val66Met polymorphism in BDNF has been associated with the development of bipolar disorder, depression, obsessive compulsive disorder, eating disorders, SZ [85], anxiety disorders, addictions, and impairments in memory and learning [86]. In modeling this mutation, it was found that the available experimentally determined structure of BDNF does not include the prodomain region containing the 66th amino acid residue. Therefore, the study involved constructing a complete three-dimensional model of the protein using the Rosetta molecular modeling algorithm [87]. Following MD analysis with GROMACS [88], PCA, and hydrogen bond analysis were conducted. Disruptions in the coordinated motions of the N-terminal region of the mutant variant were identified, along with the loss of a hydrogen bond between key amino acid residues. The region exhibiting altered dynamic fluctuations includes the pre- and prodomains, which are critical for protein sorting and secretion [89]. Defective delivery and activation of BDNF in neurons impair neuronal plasticity and synaptic transmission. These findings demonstrate how even a single amino acid substitution, without affecting the global stability of the protein, can alter its dynamics and interactions in functionally important regions.

In addition to investigating mutations within soluble and cytoplasmic proteins, biological psychiatry frequently targets ion channels and neurotransmitter receptors located on the cell surface. The physiological gating and signal transduction of these membrane complexes rely on macroscopic conformational transitions between intermediate structural states. Because these large-scale rearrangements, such as the opening of an ion pore, involve crossing high energetic barriers and occur too slowly to be captured by classical sampling, TMD is applied to evaluate how specific genetic variants alter the kinetics of these transitions [90]. The TMD approach has been applied to assess the impact of mutations in the NMDA receptor, which phenotypically manifest as developmental delay, epilepsy, SZ, ID, ASD, attention-deficit hyperactivity disorder, visual impairment, hypotonia, speech disorders, movement disorders, and microcephaly [91,92,93]. TMD enabled the simulation of the receptor’s transition from the closed to the open state, with the aim of identifying which amino acid residues converge and contribute to the stabilization of the open channel conformation, thereby facilitating the passage of calcium, sodium, and potassium ions across the membrane [94]. Simulations showed that upon activation, the side chains of p.Pro552 and p.Phe652 converge to form a favorable van der Waals contact, observed only in the open state, stabilizing the open-state pore conformation. Mutational analysis followed by MD indicated that the p.Pro552Arg substitution strengthens this contact owing to arginine’s longer side chain; however, the resulting over-stabilization leads to a loss-of-function phenotype, with the channel opening only rarely and exhibiting infrequent but prolonged open events. In contrast, the p.Phe652Val mutation perturbs the interaction geometry and weakens the contact, thereby reducing open-state stability and shortening open times. These findings lay the groundwork for interpreting these mutations in the context of epilepsy and ID [95,96].

Methodological boundaries in computational biophysics are not absolute, and the selection of a simulation technique depends on the specific structural state and energetic barriers of the physiological event rather than a rigid classification of the protein family. Even when investigating complex transmembrane assemblies that typically require biased approaches to simulate macroscopic gating, standard equilibrium simulations can capture functionally critical events if the target is already in a low-barrier state. Accordingly, in certain cases, when the ion channel is modeled in its open conformation and has a relatively short conduction pathway, thermal fluctuations inherent to classical MD simulations may be sufficient to permit spontaneous ion permeation, without the need for external electric fields or enhanced sampling techniques. MD simulations of the HCN1 channel (hyperpolarization-activated cyclic nucleotide-gated channel 1), a voltage-gated ion channel primarily permeable to K^+^ and involved in neuronal excitability, were used to assess the structural effects of the p.Gly391Asp mutation. It was found that the mutant homotetramer failed to conduct ions. The mutation introduced a negatively charged aspartate side chain at a critical site, which formed a tight electrostatic complex with cations (especially K^+^) inside the pore. This interaction physically blocked the ion conduction pathway, rendering the channel non-functional [97]. Due to the diverse computational approaches applied to similar targets, researchers frequently conduct short exploratory simulations to assess initial system behavior before initiating full-scale production runs. Additionally, these brief simulations serve as a computational screening tool to evaluate multiple alternative conformations of the same model, enabling the targeted selection of the most structurally viable or functionally relevant states [95,96].

## 4. Analysis of Post-Translational Modifications

The functioning of proteins can change significantly not only as a result of folding but also under the influence of PTMs—site-specific covalent modifications of amino acid side chains or the peptide backbone that occur after ribosomal synthesis. Protein PTMs serve as the downstream regulatory interface connecting genetic risk with cellular phenotype. There is compelling evidence that glycosylation and lipidation are impaired in the brains of patients with SZ, affecting both proteins directly involved in neurotransmission (e.g., EAAT1/2, GluA2, GABRA1/2) and the enzymes responsible for the synthesis and modification of PTMs (e.g., MGAT4A, FUT8, ZDHHC5, PPT1) [98,99,100,101]. This makes PTMs promising targets for new biomarkers and therapeutic approaches, especially given their high sensitivity to changes in cellular context and environment. However, many PTMs occur within intrinsically disordered regions or membrane microdomains, which are often experimentally intractable. MD simulations make it possible to model key PTMs, such as phosphorylation, acetylation, methylation and glycosylation [102,103]. The study of the MAP2 protein provides a compelling example of how these in silico methods can elucidate the effects of aberrant phosphorylation. MAP2 stabilizes microtubules in neuronal dendrites and regulates their structure and plasticity; under physiological conditions, it undergoes selective post-translational phosphorylation [104]. In patients with SZ, despite the preserved overall expression level of this protein, hyperphosphorylation occurs at p.Ser1782, which is associated with loss of microtubule binding, impaired dendritic spine formation, and local inhibition of protein synthesis. Secondary-structure analysis of the MD trajectories indicates that phosphorylation at p.Ser1782 destabilizes the compact β-hairpin present in wild-type MAP2, promoting its unfolding into an extended, solvent-exposed β-conformation.

Conversely, when PTMs do not precipitate large-scale conformational unfolding, but instead localize to specific interaction interfaces, end-state thermodynamic methods such as MM/GBSA are employed to quantify the resulting shifts in protein–protein or protein–ligand binding affinities [60]. Following this rationale, this approach was applied to evaluate the binding energetics of the tau protein’s R2 domain, where pathological phosphorylation, particularly at positions p.Ser289 and p.Ser293 in Alzheimer’s disease, disrupts the interaction interface, leading to a loss of microtubule stabilization and accelerated aggregation [105]. To quantitatively characterize these effects, MD simulations were performed for four variants of the R2 domain: wild type, monophosphorylated at p.Ser289 or p.Ser293, and with double modification. The resulting simulation trajectories were analyzed using MM/GBSA, consistent with the aforementioned WSAS and Interaction Entropy estimation methods, to precisely calculate the binding free energy to microtubules alongside an evaluation of the proteins’ secondary structures. Monophosphorylation at either p.Ser289 or p.Ser293 markedly weakens the binding of the tau R2 repeat to microtubules, and this effect is compounded by dual modification, which results in a near-complete loss of affinity. Quantitative binding free energy calculations confirm this observation: the binding free energy decreases. For the doubly phosphorylated peptide, the binding free energy is diminished to a negligible minimum, denoting a severely destabilized interaction. To identify the origin of this destabilization, a free energy decomposition analysis was performed. This revealed that the phosphorylated residues themselves make the most significant unfavorable contribution to the binding energy. This energetic penalty is physically manifested in a significant reduction in intermolecular contacts. As shown by interaction maps, the phosphorylated peptides exhibit a substantially lower frequency of contact with key residues on the microtubules compared to the wild-type system, thereby explaining the observed loss of binding affinity. Analysis of the secondary structure revealed that phosphorylation promotes a significant conformational shift within the R2 peptide. Specifically, the modification disrupts ordered α-helical structures, driving a conformational shift toward disordered random coils that is particularly pronounced in the middle of the sequence and at the N-terminus. By elevating the disordered content in these regions, this structural transition physically exposes the highly aggregation-prone PHF6* hexapeptide, thereby establishing a thermodynamic driving force for tau peptide aggregation [106].

Protein aggregation and phase separation of intrinsically disordered proteins are classically slow conformational processes that distinctly illustrate the computational necessity of coarse-grained modeling. To bypass the prohibitive computational costs of all-atom models, researchers employed coarse-grained MD coupled to stochastic Monte Carlo phosphorylation steps (MD/MC) to simulate massive systems comprising 200 TDP-43 chains over requisite timescales of up to 30 microseconds, which cytoplasmic clustering in associated with frontal lobe dementia (FTLD) [107,108,109]. At the monomeric level, CK1δ exhibits a 3–4-fold phosphorylation preference for the C-terminus. This pattern correlates with active-site contact frequency, driven by aromatic affinity and N-terminal electrostatic repulsion, initiating a positive feedback loop that increases binding energy and accelerates subsequent modifications. Mesoscopically, CK1δ localizes exclusively to the condensate surface, where its disordered tail acts as a penetrating physical anchor while simultaneously attenuating overall reaction rates via transient autoinhibition. Progressive surface phosphorylation generates critical electrostatic repulsion; macroscopic dissolution initiates upon modification of ~25% of available serines. Individual hyperphosphorylated chains detach after acquiring ~16 phosphate groups, driving complete droplet dissolution within 5 microseconds. These mechanics delineate two divergent hypotheses for FTLD pathogenesis: a compensatory cellular defense mechanism solubilizing pre-existing neurotoxic aggregates, or a structural failure where hyperphosphorylation disrupts critical α-helices, precipitating cytoplasmic mislocalization.

The examples discussed above represent only a fraction of PTMs linked to pathology. Like mutations in well-characterized neuroreceptors, such as GPCRs, AMPA, NMDA, and GABA receptors, PTMs are fundamental to the life cycle and signal regulation of synaptic membrane proteins [110,111]. Physiologically, PTMs make GPCR signaling precise and context-dependent. These modifications modulate synaptic efficacy by regulating receptor stabilization, allosteric transitions, and vesicular trafficking at the postsynaptic density. In pathological states, however, aberrant PTMs disrupt not only receptor activation but also protein folding, membrane targeting, and degradation. This dysregulation ultimately alters neurophysiology and pharmacological sensitivity. Demonstrating this effect, a hybrid simulation of the human dopamine transporter under distinct phosphorylation patterns resolved its transport states, free-energy differences, and transition probabilities. These ensemble-level changes correlate directly with experimental variations in dopamine uptake. Specifically, the NP-333 condition biased sampling toward intermediate states and reduced access to uptake-competent conformations. These findings show that post-translational modulation directly alters the functional cycling of monoaminergic transporters [112]. Because not all sequence variants and PTM events result in measurable functional consequences, in silico analysis provides an important filter for prioritizing those most likely to alter protein structure, conformational equilibria, or transport dynamics. However, the utility of MD remains scale-dependent: although it can resolve local structural perturbations and protein-state transitions, the spatiotemporal complexity of maturation and trafficking through the endoplasmic reticulum and Golgi apparatus still lies largely beyond the direct reach of conventional simulations [113].

## 5. Psychopharmacological Drug Development

The treatment of psychiatric disorders is frequently complicated by resistant forms, which emerge from underlying genetic heterogeneity [114,115]. Although antipsychotic medications remain the standard therapeutic approach for psychosis, a substantial proportion of patients show only a limited response. Incomplete symptomatic recovery is observed in 19% to 89% of cases, while rates of social and occupational dysfunction remain persistently high, reaching up to 86%. This marked variance in clinical outcomes demonstrates a compelling need for novel pharmacotherapeutic strategies [116,117]. Furthermore, psychopharmacology faces severe financial barriers during drug development and market approval. The average cost of developing a single pharmaceutical agent is estimated at $879.3 million in 2018 USD, accounting for direct expenditures, capitalized costs, and failed projects. According to data from the FDA and ClinicalTrials.gov, Phase 3 clinical trials consistently represent the costliest stage [118]. Therefore, only drug candidates demonstrating the highest therapeutic potential and a well-substantiated mechanism of action should advance to this phase. Applying computational modeling prior to clinical trials offers a method to reduce these development costs by as much as 50%, primarily by improving compound prioritization and minimizing late-stage attrition [119]. This strategy is particularly relevant in psychiatry, where molecular modeling allows researchers to integrate individual mutational profiles, thereby accelerating the development of targeted compounds within a personalized medicine framework.

Molecular docking drives virtual screening and rational ligand design by modeling how potential compounds interact with CNS targets, accelerating the discovery of selective psychoactive agents. Because of the computational burden of MD, it is typically reserved for the end of these workflows to refine and validate a small set of top-ranked docking hits. Rigid docking, a computationally efficient method, predicts ligand–receptor interactions by assuming fixed conformations for both molecules [120,121]. This makes it ideal for scanning large compound or receptor libraries against structurally well-defined targets, such as dopamine D2 and endocannabinoid receptors in SZ or in psilocybin-mediated suicide prevention [122,123]. Applying rigid docking to evaluate quetiapine, olanzapine, and clozapine against over 220 AlphaFold-predicted human receptors revealed distinct binding profiles for each drug. These affinities extend beyond classical neuropharmacological targets, such as 5-HT1BR for olanzapine, to include immunological and metabolic receptors like CB2, NPYR4, and CCR5. Subsequent analysis indicated that the immunosuppressants cyclosporine A and everolimus exhibit high affinity for these same targets, suggesting their potential as structural scaffolds for novel antipsychotic therapies [124].

In realistic biomolecular systems, ligand binding is frequently associated with conformational adjustments of the active site, whereby the protein structurally adapts to the incoming molecule, effectively “enclosing” it. Therefore, to account for the flexibility of both the ligand and the receptor, flexible or induced-fit docking approaches are applied [125]. Applying these dynamic docking principles to neuropharmacology has proven essential for elucidating intricate ligand–receptor interactions, particularly those involving atypical psychoactive agents. MDMA (3,4-methylenedioxy-methamphetamine) and its close analogues (MDA, MBDB, and MDAI) belong to the class of entactogens, which are psychoactive substances capable of inducing empathogenic and pro-social states. Unlike classical psychedelics, they do not typically produce pronounced hallucinations and are being considered as promising agents for the treatment of post-traumatic stress disorder [126,127], anxiety disorders [128], personality disorders [129], and depression [130,131]. The primary molecular target of entactogenic compounds, including MDMA, is the human serotonin transporter (hSERT). Through its interaction with this protein, MDMA induces the release of serotonin while concurrently inhibiting its reuptake. This dual action originates from the ligand’s positional and conformational dynamics within the orthosteric binding site (S1). The binding of MDMA within this site is anchored by several key interactions: a primary anchor is the formation of a salt bridge between MDMA’s methylammonium group and the amino acid residue Asp98, a highly conserved interaction for both substrates and inhibitors. Furthermore, the molecule engages in a complex interaction with the critical residue Tyr95, concurrently forming both a hydrogen bond and a cation-π interaction [132]. The benzodioxole ring of MDMA is further stabilized through edge-to-face π-π stacking interactions with gating residues, including p.Tyr176 and p.Phe341. Unlike classic inhibitors that adopt a fixed position, MDMA demonstrates significant conformational mobility, occupying multiple energetically favorable, alternative positions within the binding pocket. This dynamic behavior is consistent with MDMA functioning as a transported substrate rather than a static blocker; it follows the transporter’s conformational cycle and passes through the inward-open gate instead of stabilizing it [132]. This mechanistic framework explains MDMA’s potent ability to elevate extracellular serotonin levels, which in turn underlies its characteristic psychopharmacological effects, including enhanced empathy, emotional openness, and reduced fear.

**Figure 3:**
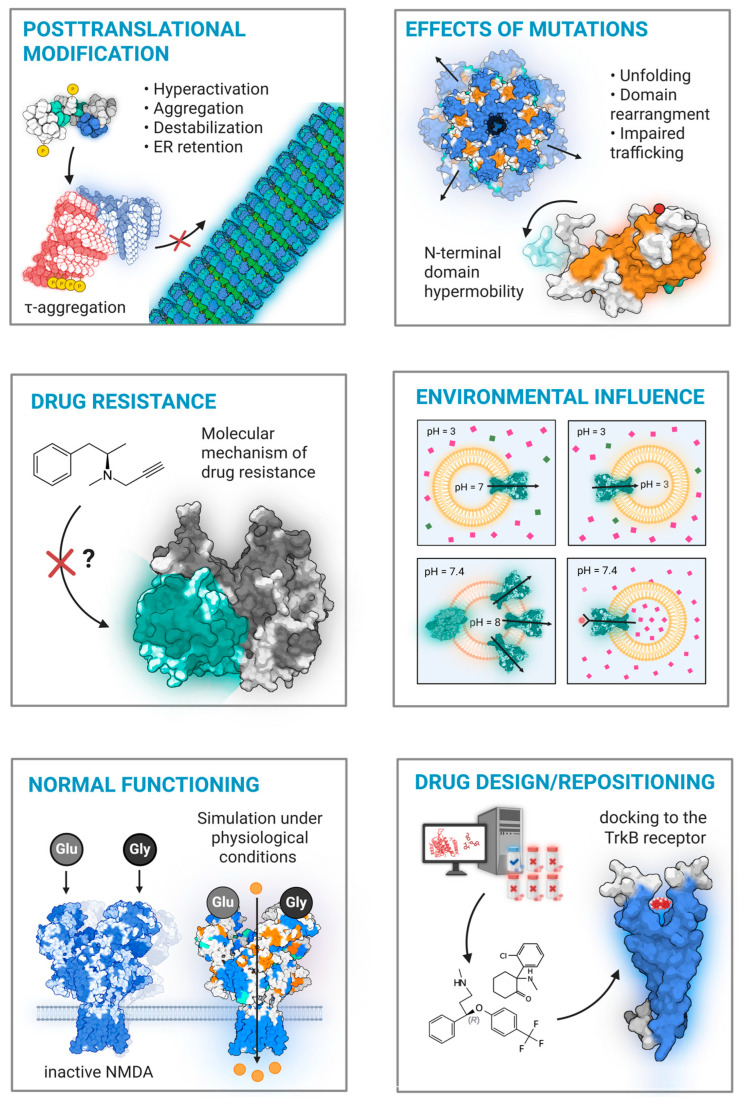
**The scope of MD simulations in psychiatric research**. MD provides insights into the fundamental processes underlying psychiatric disorders. These applications include investigating the consequences of post-translational modifications that drive protein aggregation and destabilization; determining the structural and functional effects of mutations that cause domain rearrangement and impaired trafficking; and aiding the rational design of therapeutics. Furthermore, MD can elucidate molecular mechanisms of drug resistance, assess the impact of environmental factors like pH on protein function, and characterize normal receptor dynamics to establish a baseline for studying disease states. Created with BioRender, https://tinyurl.com/35f4zwwu (accessed on 12 March 2026).

MD simulations offer a robust methodology for testing hypotheses regarding the mechanisms of drug action and for elucidating the basis of therapeutic resistance (Figure 3). Many common antidepressants, such as fluoxetine, imipramine, and ketamine, are amphiphilic compounds. This property allows them to interact with the lipid phase of cellular membranes and to accumulate in cholesterol-rich lipid rafts, which function as critical hubs for cellular signaling systems. It is well-established that several antidepressants upregulate the expression and activity of BDNF and its receptor, tropomyosin receptor kinase B (TRKB), and that the blockade of TRKB signaling nullifies their behavioral effects. Historically, the effect of antidepressants on the TRKB receptor was considered indirect and mediated by primary targets like the serotonin transporter for SSRIs and NMDA receptors for ketamine [133,134]. However, MD and docking simulations demonstrate that membrane-bound TRKB dimerizes into a cross-like structure, allowing fluoxetine to insert into an inter-helical cleft and thermodynamically stabilize the receptor. Point mutations within the binding pocket (p.Tyr433Phe, p.Val437Ala, and p.Ser440Ala) support this direct-binding mechanism of antidepressant and receptor; they significantly reduce fluoxetine’s residence time and prevent the drug from maintaining TRKB in an active conformation [135].

Furthermore, this study demonstrates the utility of MD in evaluating protein behavior across different microenvironments. Simulations using various neuronal membrane models revealed that TRKB dimerization depends heavily on lipid composition. The receptor reaches optimal activity at a cholesterol concentration of approximately 20%. At this level, lipid molecules directly participate in the interaction, forming an enclosed pocket that secures fluoxetine through lipid-mediated binding. While fluoxetine stabilizes the active receptor structure, cholesterol concentrations above or below this 20% optimum induce conformational distortions. These distortions reduce TRKB sensitivity to BDNF and attenuate downstream signaling [135]. This cholesterol dependence explains observed developmental differences in the brain. Childhood synapses contain relatively low cholesterol levels [136], maintaining high TRKB surface expression and robust neuroplasticity. In contrast, mature adult synapses possess higher cholesterol concentrations, which often leaves TRKB inactive and sequestered within intracellular vesicles. By stabilizing the active TRKB dimer, antidepressants temporarily restore a state of elevated neuroplasticity. This pharmacological shift creates a biological window that facilitates targeted psychotherapeutic interventions for conditions such as depression and anxiety disorders [137,138].

Another clinically relevant example that helps explain antidepressant nonresponse and apparent treatment resistance in routine practice is pharmacokinetic variability, in which functional genetic variation in the CYP2D6 drug-metabolizing enzyme can shift antidepressant exposure even under standard dosing [139]. Among variants linked to antidepressant outcomes in major depressive disorder, the CYP2D6 rs1065852 polymorphism that corresponds to p.Pro34Ser has been described as functionally significant, and molecular dynamics simulations indicate that this change reduces the structural flexibility of the loop forming part of the substrate access channel, thereby impairing catalytic function. Because CYP2D6 contributes to the hepatic metabolism of many antidepressants, reduced activity in carriers of this variant can plausibly alter drug plasma concentrations and contribute to inconsistent clinical responses, providing a mechanistic bridge between genotype and the well-documented interindividual variability in antidepressant efficacy and supporting CYP2D6 genotyping as a rational component of personalized pharmacotherapy in major depressive disorder [140]. This specific CYP2D6 example perfectly illustrates why researchers have to combine different computational scales. Usually, finding a variant like this starts from the top down. Systems biology and statistical genetics operate at a massive scale, using multi-omics and network models to flag these connections in the first place. But while these methods excel at generating hypotheses and explaining high-level phenotypes like synaptic signaling, they completely bypass the precise molecular architecture of specific receptors [141,142,143]. Resolving how a mutation like the CYP2D6 loop alteration actually reshapes a protein requires atomistic resolution. Docking and virtual screening offer a high-throughput starting point, generating plausible binding poses and ranking compounds at scale. However, because they prioritize computational speed over thermodynamic rigor, their outputs function as structural hypotheses rather than precise affinity measurements. MD then steps in as the final mechanistic check. MD takes a shortlist of those variants or ligands and simulates the actual physics, tracking water molecules, ions, and side-chain packing [144]. This step is particularly critical in psychiatry because docking alone misses the subtle water-network rewiring and allosteric shifts that drive receptors into hyperactive or desensitized states [143,144]. While MD is still limited by simulation timescales and force-field accuracy, it delivers concrete, testable predictions that can be fed back upstream to tell systems models which variants are functionally relevant. The most rigorous workflow links all these pieces into a continuous loop. Systems biology maps the pathways, docking screens the compounds, MD tests the underlying physics, and lab experiments validate the whole pipeline [9,141,144,145].

## 6. Conclusions and Future Perspectives

Over the past 25 years, the scale and duration of MD simulations have increased significantly, driven by parallel advances in computational hardware and algorithmic innovation [103]. This progress is exemplified by the significant extension of accessible timescales. In the early 2000s, typical all-atom simulations of proteins were limited to tens or hundreds of nanoseconds. However, following the widespread adoption of GPU acceleration in modeling software since the 2010s, the achievable simulation length for many researchers has extended into the microsecond-to-millisecond range [146]. This leap in computational power has enabled the detailed investigation of increasingly complex systems, as seen in numerous studies, we have shown, of protein interactions and membrane protein dynamics.

MD proves to be a valuable tool for both investigating the structure and function of biological systems, as well as for the clinical assessment of the significance of mutations in patients with neuropsychiatric disorders. The dynamic development of methods for modeling biological systems, increasing their duration, complexity, and accuracy, opens up the prospect of studying large protein ensembles that are important for neuronal signal transmission and synaptogenesis, including in the context of pathological nervous system function. Furthermore, while such long physiological timescales remain challenging for routine all-atom MD, coarse-grained and multiscale frameworks are making them increasingly accessible. This shift may soon enable approximate reconstructions of whole-cell proteome dynamics [12], as demonstrated by recent atomistically resolved models of bacterial cytoplasm that capture near-cellular macromolecular crowding [147].

One of the most widely used tools for this multiscale strategy is the Martini force field. By reducing atomic detail into broader interaction sites, it enables simulations of multi-protein and multi-lipid assemblies at spatiotemporal scales that are otherwise impractical [148]. For biological psychiatry, this makes it possible to move beyond static structural models and test how lipid composition and membrane crowding reshape nanoscale synaptic organization, driving processes like GPCR clustering and transporter oligomerization [148,149].

This gain in scale, however, comes at the cost of reduced structural resolution. Proteins often require elastic-network restraints to preserve their fold, making any inferred conformational transitions more model-dependent [150]. In addition, while earlier Martini parameterizations could over-stabilize protein–protein associations and promote artificial aggregation, Martini 3 substantially improves this balance and reduces such artifacts [149,151]. Accordingly, coarse-grained simulations are best viewed as a high-throughput exploratory layer for identifying large-scale organizational trends. Once these trends are established, they can be followed by targeted all-atom refinement when precise drug-binding energetics, ion-coordination motifs, or residue-level contacts are required.

The integration of trajectory-trained generative models shifts MD workflows from brute-force exploration to the rapid generation of structural ensembles, utilizing MD primarily for validation. For example, generative adversarial networks trained on MD data can directly predict sequence-dependent conformational ensembles for intrinsically disordered proteins, including previously unseen sequences, at a negligible computational cost [152,153]. Similarly, transformer-based models trained on protein–protein complexes can predict novel, long-timescale conformations that are computationally inaccessible via standard MD, utilizing attention mechanisms to identify key interfacial amino acid residues that drive these macro-dynamics [154]. However, because independently generated conformations lack kinetic information and inevitably inherit the artifacts of the underlying simulations, classical MD remains necessary. The field is progressing toward a hybrid methodology: AI efficiently proposes diverse candidate microstates, while MD applies physical constraints, filters structural artifacts, and extracts reliable thermodynamic and kinetic properties [155,156].

Contemporary molecular dynamics studies in neuropsychiatry are characterized by focused, yet often disparate, investigations into the behavior of individual proteins implicated in the pathogenesis of entire clusters of related disorders. The systematic accumulation of such high-resolution dynamic data is poised to transition the field from correlational observations to the formulation of specific, mechanistically grounded theories of mental illness. Ultimately, this growing body of molecular-level evidence will serve as an additional interpretive layer. It will not only facilitate a more comprehensive functional annotation of the vast genetic data emerging from large-scale biobanks and international consortia but will also guide the rational design of targeted therapeutic interventions.

## Data Availability

No new data were created or analyzed in this study. Data sharing is not applicable to this article.
